# Antianginal effects of empagliflozin in patients with type 2 diabetes and refractory angina; a randomized, double‐blind placebo‐controlled trial (EMPT‐ANGINA Trial)

**DOI:** 10.1002/clc.24158

**Published:** 2023-09-18

**Authors:** Mohammad Hadi Mansouri, Pejman Mansouri, Masoumeh Sadeghi, Seyedeh Melika Hashemi, Alireza Khosravi, Mohaddeseh Behjati, Javad Shahabi, Asieh Mansouri, Reihaneh Zavar, Afshin Amirpour, Hamid Sanei, Nizal Sarrafzadegan

**Affiliations:** ^1^ Hypertension Research Center, Cardiovascular Research Institute Isfahan University of Medical Sciences Isfahan Iran; ^2^ Tehran Heart Center Tehran University of Medical Sciences Tehran Iran; ^3^ Cardiac Rehabilitation Research Center, Cardiovascular Research Institute Isfahan University of Medical Sciences Isfahan Iran; ^4^ Heart Failure Research Center, Cardiovascular Research Institute Isfahan University of Medical Sciences Isfahan Iran; ^5^ Isfahan Cardiovascular Research Center, Cardiovascular Research Institute Isfahan University of Medical Sciences Isfahan Iran

**Keywords:** angina pectoris, clinical trial, coronary artery disease, diabetes, empagliflozin, refractory, stable angina

## Abstract

**Introduction:**

Sodium–glucose cotransporter 2 (SGLT2) inhibitors are emerging antidiabetic agents with various potential cardiovascular benefits. The EMPT‐ANGINA trial examined the effect of empagliflozin on the angina burden in those with concurrent type 2 diabetes mellitus (T2DM) and refractory angina (RA).

**Method:**

In this 8‐week, double‐blind, randomized, placebo‐controlled trial, 75 patients with T2DM and RA were randomly assigned to one of two groups: empagliflozin (*n* = 37) and placebo (*n* = 38). The primary outcome was an improvement in angina, which was assessed by the Seattle Angina Questionnaire (SAQ). The secondary outcomes of this study included alterations in the SAQ domains and exercise test components.

**Results:**

The mean age of individuals in the empagliflozin and placebo groups was 67.46 ± 9.4 and 65.47 ± 7.0 years, respectively (*p* = .304). Patients who received empagliflozin showed a significant improvement in both the primary endpoint, which was the SAQ Summary Score (192.73 ± 20.70 vs. 224 ± 25.36, *p* < .001) and the secondary endpoints. Exercise test components, including treadmill exercise duration, time till angina, 1 mm ST‐segment depression onset, and heart rate (HR) recovery, were all significantly improved in the empagliflozin group. This positive impact was reached with no clinically significant changes in resting and exertion HR or blood pressure. There were no significant side effects in the empagliflozin group (*p* = .125).

**Conclusion:**

Empagliflozin can be safely added as a metabolic modulator agent to existing antianginal medications in individuals with concurrent T2DM and RA to reduce angina symptoms and enhance exercise capacity with minimal side effects.

## Graphical abstract

The EMPT‐ANGINA randomized trial evaluated the effect of empagliflozin, a sodium–glucose cotransporter 2 (SGLT2) inhibitor, on angina symptoms and exercise capacity in patients with both type 2 diabetes mellitus (T2DM) and refractory angina (RA). Patients were divided into two groups: one received empagliflozin (*n* = 37), and the other received a placebo (*n* = 38). The primary outcome, assessed by the Seattle Angina Questionnaire Summary Score, showed significant improvement in the empagliflozin group. Secondary outcomes, including various exercise test components, also improved significantly in the empagliflozin group. The study concluded that adding empagliflozin to routine anti‐hyperglycemic therapies in patients with T2DM and RA could significantly reduce angina symptoms and improve exercise capacity with minimal side effects.

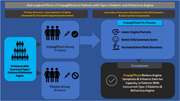

## 1 INTRODUCTION

Atherosclerotic cardiovascular disease (ASCVD) remains one of the leading causes of mortality and morbidity in patients with type 2 diabetes mellitus (T2DM).^1^, ^2^ Reports indicate that 25%–45% of patients with concurrent coronary artery disease (CAD) and T2DM experience stable angina, leading to limitations in physical activity and impaired quality of life (QOL).^3^, ^4^

The term “refractory angina” (RA) refers to the chronicity of symptoms (≥3 months in duration) and failure of symptom control with a combination of optimal medical therapy and revascularization methods.^5^, ^6^, ^7^ Between 5% and 10% of patients who undergo cardiac catheterization have RA. However, there is no specific data on how this condition affects diabetic patients.^8^

Traditional treatments for angina and myocardial ischemia are based on increasing coronary blood flow, increasing blood oxygen‐carrying capacity, and decreasing oxygen consumption; however, more recent treatments compromise agents that alter the metabolism of myocytes (Trimetazidine and Perhexiline), inhibition channels (Ranolazine), and use invasive (percutaneous myocardial laser revascularization) and noninvasive (enhanced external counterpulsation [EECP]) techniques.^9^ Ranolazine, one of the newest and most well‐known antianginal medications, which has a fundamentally different mechanism of action than empagliflozin, has been studied in patients with angina. In a study conducted by Kosiborod et al. on patients with concomitant T2DM, CAD, and chronic stable angina who remain symptomatic despite antianginal medication (TERISA trial), weekly angina frequency (AF) was considerably reduced with ranolazine compared to placebo.^5^

The effectiveness of antianginal drugs in patients who have both stable angina and T2DM is not well studied.^6^ Some antianginal medications, such as beta‐blockers and calcium channel blockers, can worsen the glycemic control of these patients, so it is important to choose anti‐anginal drugs that have a neutral or positive impact on them.^10^, ^11^ Some non‐antianginal agents, such as thiazolidinediones and metformin, have been evaluated for angina treatment among these patients.^3^ Etomoxir, carnitine palmitoyl‐transferase 1 inhibitor, was initially introduced as an antidiabetic agent with potential antianginal properties suggested in some studies.^12^ One animal study showed that canagliflozin decreased myocardial infarct size in both diabetic and nondiabetic rats, independent of glucose concentration at the time of ischemia or reperfusion injury over a 4‐week period.^13^ Moreover, another study revealed some coronary vasodilation properties of sodium–glucose cotransporter 2 (SGLT2) inhibitors (empagliflozin and canagliflozin) in healthy mouse hearts.^14^

Large randomized controlled trials on SGLT2 inhibitors in T2DM have shown remarkable benefits on cardiovascular and renal outcomes in individuals with established ASCVDs.^15^, ^16^, ^17^, ^18^, ^19^, ^20^, ^21^, ^22^ Recent guidelines recommend administering SGLT2 inhibitors as second‐line therapy after Metformin, especially in patients with CVD.^23^, ^24^ Empagliflozin, an SGLT2 inhibitor, switches energy substrate preference from fatty acids (FAs) to glucose, reduces lactate production, and improves myocardial work efficiency and function.^25^, ^26^

Due to the possible antianginal effect of SGLT2 inhibitors, we conducted a double‐blind, placebo‐controlled, randomized trial to evaluate the potential impact of empagliflozin on angina burden and exercise capacity in patients with both RA and T2DM, regardless of its glucose‐lowering effects.

## 2 MATERIALS AND METHODS

### 2.1 Study design

In the EMP‐ANGIN study (URL: http://www.clinicaltrials.gov, registration number NCT04143321), patients with concurrent T2DM and RA who were using conventional antianginal medications were randomly assigned to the empagliflozin or placebo group in a double‐blinded, placebo‐controlled trial between November 2019 and January 2021. The investigation was carried out in two tertiary cardiac facilities in Isfahan, Iran. The ethics committee of Isfahan University of Medical Sciences (IUMS) reviewed and approved the study protocol. The primary investigator explained the study's aim in detail to each eligible participant before starting it, and all the individuals who joined the study could ask any questions they had.

### 2.2 Study population

Participants were included if they were aged over 18 with concurrent T2DM and CADs. Moreover, key inclusion criteria were as follows: (1) baseline AF score of ≤90 on SAQ, (2) treatment with at least three antianginal medications for more than 3 months, (3) unsuitable candidates for revascularization procedures (either percutaneous coronary intervention or coronary artery bypass grafting), (4) reproducible angina with concomitant ischemic ST‐segment depression of at least 1 mm on the electrocardiogram (ECG), (5) restricted treadmill testing exercise capacity (3–9 min on a modified Bruce protocol) at baseline.

Key exclusion criteria initially included: (1) New York Heart Association (NYHA) functional class of III–IV, (2) presence of heart failure symptoms, (3) an acute coronary syndrome in the past 2 months, (4) any coronary revascularization during the study period, (5) the occurrence of stroke or transient ischemic attack within 6 months of screening, (6) uncontrolled hypertension, (7) clinically significant hepatic or renal impairment, (8) prior treatment with empagliflozin, (9) recent use of invasive treatments for RA (including the EECP), extracorporeal shockwave therapy, coronary sinus reducer implantation, spinal cord stimulation, or sympathectomy), (10) any relative or absolute contraindications for the exercise test, (11) specific conditions that may preclude the accurate interpretation of ECG (such as left bundle branch block, resting ST depression (STD) more than 1 mm, pre‐excitation rhythm abnormalities or usage of Digoxin).

### 2.3 Interventions, clinical measurements, and data collection

Patients who met our inclusion criteria were recruited from outpatient visits at comprehensive diabetic care and cardiology clinics. An informed consent form was signed by each person before the patient was enrolled. All Patients had baseline ECG, heart rate (HR), and blood pressure (BP) measurements recorded, as well as height and weight. Also, patients completed the Seattle Angina Questionnaire (SAQ). Patients were assessed at study visits for major outcomes, including BP, HR, and exercise stress test with a modified Bruce protocol at the time of enrollment and 8 weeks later.

After enrollment, the study consisted of two consecutive phases. The first was a 4‐week run‐in period for extended screening. Cases with SAQ <90 were kept under remote observation for about four consecutive weeks. If SAQ remained less than 90, the participant entered the randomization process. Eligible subjects were randomized to receive either a placebo or empagliflozin (25 mg/day) for 8 weeks. Prior prescribed antianginal drugs were continued for participants in both arms during the study period. Both investigators and participants remained blinded to the treatment arms until the completion of the trial, and data collection was performed by a physician. Participants were followed each week with telephone calls for assurance of proper daily pill consumption and to report possible side effects.

### 2.4 Definitions

The SAQ is a 19‐item, self‐administered questionnaire that has been validated for use in patients with CADs. The questionnaire is divided into several categories, including angina pectoris frequency, physical limitations (PL), angina stability (AS), treatment satisfaction (TS), and QOL. For each of these five categories, the scores range from 0 (most symptomatic) to 100 (least symptomatic).^27^, ^28^ All participants completed the SAQ, and in the presence of illiterate subjects, the questionnaire was filled out with the help of their accompanies.

Ischemia in exercise‐induced ECG was defined as a newly developed horizontal or down‐sloping ST‐segment depression of ≥1 mm at 60–80 ms after J point. STD was defined as an additional ST‐segment depression of at least 1 mm below the resting value.^29^, ^30^ Postexercise heart rate recovery (HRR) was described as a clinical indicator of abnormal cardiac autonomic function. Abnormal HRR was defined as a postexercise decrease in HR of less than 12 beats per minute after 1 min or less than 18 beats per minute after 1 min with immediate cessation of movement into either supine or sitting position.^31^, ^32^

### 2.5 Study endpoints

The primary endpoint of the study was the improvement in angina symptoms, function, and patient's QOL assessed by the SAQ Summary Score (SS). The secondary outcome of this study was any alterations from the baseline of SAQ domains, including AF, PL, AS, TS, QOL, and also alternation in exercise test components, including treadmill exercise duration, STD, time to angina onset, and the mean HRR before study initiation and 8 weeks afterward.

### 2.6 Statistical analysis

Quantitative and qualitative variables were reported as mean ± standard deviation (or mean ± standard error of the mean for adjusted means) and number (percentage), respectively. The normality assumption for continuous variables was checked using the skewness test and Q–Q plots. Baseline characteristics and measurements of the participants were compared using an independent samples *t*‐test (or Mann–Whitney *U* test where appropriate) for continuous variables and Pearson's *χ* ^2^ test (or Fisher's exact test, as applicable) for categorical variables. Paired *t*‐test or Wilcoxon signed‐rank test was performed for within‐group comparisons. Mean changes (95% confidence interval [CI]) of endpoints (follow‐up minus baseline) were shown in the bar chart and compared using the independent sample *t*‐test or Mann–Whitney *U* test. Furthermore, generalized estimation equations were performed to detect any differences between the two groups at the end of the study and after adjustment for baseline values. Statistical analyses were performed using the Statistical Package for the Social Sciences version 25 (SPSS Inc.), and *p* < .05 was considered statistically significant.

## 3 RESULTS

### 3.1 Trial population

From November 2019 to January 2021, a total of 935 patients were screened and 86 patients were equally and randomly allocated to either empagliflozin or the placebo group. During the time of the study, 11 individuals (empagliflozin group: 6, placebo group: 5) were lost to follow‐up, and eventually, a total of 75 patients were included in the analyses. A detailed explanation of screening, randomization, and follow‐up is shown in Figure S1. Data on the general baseline characteristics of the study population are shown in Table 1.

**Table 1:** Comparison of baseline characteristics between treatment groups.

	Empagliflozin (*n* = 37)	Placebo (*n* = 38)	*p* Value
Age (years)	67.46 ± 9.4	65.47 ± 7.0	.304^a^
Male (%)	27 (73.0)	23 (60.5)	.253^b^
BMI (kg/m^2^)	27.03 ± 2.8	28.29 ± 3.1	.067^a^
HR	78.81 ± 11.51	73.74 ± 11.77	.063^a^
SBP	146.27 ± 25.41	133.95 ± 30.84	.063^a^
DBP	85.24 ± 13.65	83.08 ± 15.59	.325^a^
HbA1c	7.43 ± 0.58	7.42 ± 0.59	.934^a^
SAQ	241.35 ± 25.17	230.39 ± 19.88	.040^a^
PL	47.68 ± 7.93	40.58 ± 7.24	.001^c^
AS	48.92 ± 7.18	46.97 ± 7.16	.244^a^
AF	48.86 ± 8.98	47.63 ± 8.90	.552^a^
TS	47.27 ± 7.40	48.84 ± 8.93	.410^a^
QOL	48.62 ± 8.15	46.37 ± 7.82	.226^a^
TED	339.65 ± 96.51	340.76 ± 60.07	.952^a^
TAO	314.11 ± 87.49	316.97 ± 57.28	.868^a^
STD	293.49 ± 82.63	309.79 ± 64.81	.514^a^
HRR	9.81 ± 4.10	7.68 ± 2.83	.027^a^
Smoking (%)	7 (18.9)	9 (23.7)	.615^b^
Hypertension (%)	14 (37.8)	14 (36.8)	.929^b^
Prior MI (%)	27 (73.0)	22 (59.5)	.219^b^
Prior CABG (%)	6 (16.2)	8 (21.1)	.591^b^
Prior PCI (%)	14 (37.8)	14 (36.8)	.929^b^
Prior Stroke (%)	2 (5.4)	3 (7.9)	>.999^d^
CHF (%)			
No	25 (67.6)	25 (65.8)	.974^b^
NYHA Class I	5 (13.5)	5 (13.2)	
NYHA Class II	7 (18.9)	8 (21.1)	
Background medications			
BB (%)	32 (86.5)	31 (81.6)	.562^b^
Aspirin (%)	35 (94.6)	35 (92.1)	>.999^d^
Nitrate (%)	35(94.6)	37(97.4)	.615^d^
ACE inhibitor (%)	20 (54.1)	16 (42.1)	.300^b^
ARB (%)	12 (32.4)	10 (26.3)	.561^b^
PPI (%)	11 (29.7)	15 (39.5)	.399^d^
CCB (%)	28 (75.6)	26 (68.4)	.725^b^
Statin (%)	35 (94.6)	37 (97.4)	.615^d^
Nicorandil	8 (21.6)	6 (15.7)	.768^d^
Ranolazine	23 (62.1)	21 (55.3)	.896^b^
Ivabradine	2 (5.4)	3 (7.9)	>.99d^d^

*Note*: Data are shown as frequency (percentage) or mean ± SD.

Abbreviations: ACE, angiotensin‐converting enzyme; AF, angina frequency; ARB, angiotensin II receptor blockers; AS, angina stability; BB, beta blocker; BMI, body mass index; CABG, coronary artery bypass grafting; CCB, calcium channel blocker; CHF, congestive heart failure; HRR, heart rate recovery; MI, myocardial infarction; NYHA: New York Heart Association; PCI, percutaneous coronary intervention; PL, physical limitation; PPI, proton pump inhibitor; QOL, quality of life; STD, ST depression; TAO, time to angina onset; TED, treadmill exercise duration; TS, treatment satisfaction.

^a^ Obtained from independent sample *t*‐test.

^b^ Obtained from *χ* ^2^ test.

^c^ Obtained from Mann–Whitney *U* test.

^d^ Obtained from Fisher exact test using Monte Carlo simulations.

## 4 OUTCOMES AND ESTIMATIONS

As shown in Table 2, the primary outcome, SS, improved significantly in the empagliflozin group after the prescription of empagliflozin compared with the baseline. SS was higher in the empagliflozin group after adjustment for baseline values (228.01 ± 1.20 in empagliflozin vs. 185.87 ± 1.01 in placebo, *p* < .001).

**Table 2:** Comparison means of endpoints before and after intervention within treatment groups.

	Empagliflozin (*n* = 37)			Placebo (*n* = 38)			Between group comparisona^a^		
	Baseline	Follow‐up	*p* Value	Baseline	Follow‐up	*p* Value	Empagliflozin	Placebo	*p* Value
HR	78.81 ± 11.52	72.86 ± 10.85	<.001^b^	73.74 ± 11.77	71.79 ± 11.18	.164^b^	71.14 ± 1.34	73.47 ± 1.31	.240
SBP	146.27 ± 25.41	144.05 ± 25.38	.399^b^	133.95 ± 30.84	134.97 ± 26.07	.689^b^	139.31 ± 2.47	139.59 ± 2.14	.934
DBP	85.24 ± 13.65	80.00 ± 13.75	.003^b^	83.08 ± 15.59	78.74 ± 14.01	.031^c^	78.04 ± 1.01	78.42 ± 1.02	.855^d^
HbA1c	7.43 ± 0.58	7.20 ± 0.59	<.001^b^	7.42 ± 0.59	7.43 ± 0.60	.581^b^	7.19 ± 0.01	7.29 ± 0.02	.355
PL	47.68 ± 7.93	50.38 ± 8.98	<.001^b^	40.58 ± 7.23	41.76 ± 8.25	.102^c^	45.88 ± 1.01	44.32 ± 1.02	.148^d^
AS	48.92 ± 7.2	56.27 ± 11.3	<.001^b^	46.97 ± 7.2	48.58 ± 7.8	.035^b^	55.43 ± 1.59	49.39 ± 0.73	.001
AF	48.86 ± 9.0	63.19 ± 11.6	<.001^c^	47.63 ± 8.9	47.92 ± 9.2	.581^c^	61.81 ± 1.03	47.23 ± 1.03	<.001^d^
TS	47.27 ± 7.4	54.16 ± 9.6	<.001^b^	48.84 ± 8.9	49.18 ± 9.9	.852^b^	54.47 ± 1.40	48.89 ± 1.55	.008
QOL	48.62 ± 8.1	56.86 ± 9.3	<.001^b^	46.37 ± 7.8	50.95 ± 9.9	.007^b^	56.44 ± 1.58	51.35 ± 1.46	.022
SS	192.73 ± 20.70	224.00 ± 25.36	<.001^c^	184.03 ± 20.3	187.45 ± 21.2	.218^b^	228.01 ± 1.20	185.87 ± 1.01	<.001^d^
TED	339.65 ± 96.5	384.11 ± 88.5	<.001^b^	340.76 ± 60.1	337.84 ± 57.6	.358^b^	384.62 ± 2.74	337.35 ± 2.97	<.001
TAO	314.11 ± 87.5	345.14 ± 82.5	.001^b^	316.97 ± 57.3	313.11 ± 61.5	.104^b^	346.38 ± 7.87	311.89 ± 2.85	<.001
STD	293.49 ± 82.6	316.51 ± 80.0	<.001^b^	309.79 ± 64.8	306.55 ± 67.0	.282^b^	324.21 ± 5.01	299.06 ± 2.94	<.001
HRR	9.81 ± 4.1	16.16 ± 6.2	<.001^b^	7.68 ± 2.8	8.18 ± 3.1	.342^b^	15.34 ± 0.82	8.98 ± 0.48	<.001

Abbreviations: AF, angina frequency; AS, angina stability; DBP, diastolic blood pressure; GEE, generalized estimation equation; HR, heart rate; HRR: heart rate recovery; PL, physical limitation; QOL, quality of life; SBP, systolic blood pressure; STD, ST depression; TAO, time to angina onset; TED, treadmill exercise duration; TS, treatment satisfaction.

^a^ Follow‐up mean ± SE of endpoints and measurements after adjustment for baseline values.

^b^ Obtained from paired sample *t*‐test.

^c^ Obtained from Wilcoxon signed‐rank test.

^d^ Logarithmic transformation was performed to assess the normality assumption. Anti‐log of transformed values reported.

**Figure 1:**
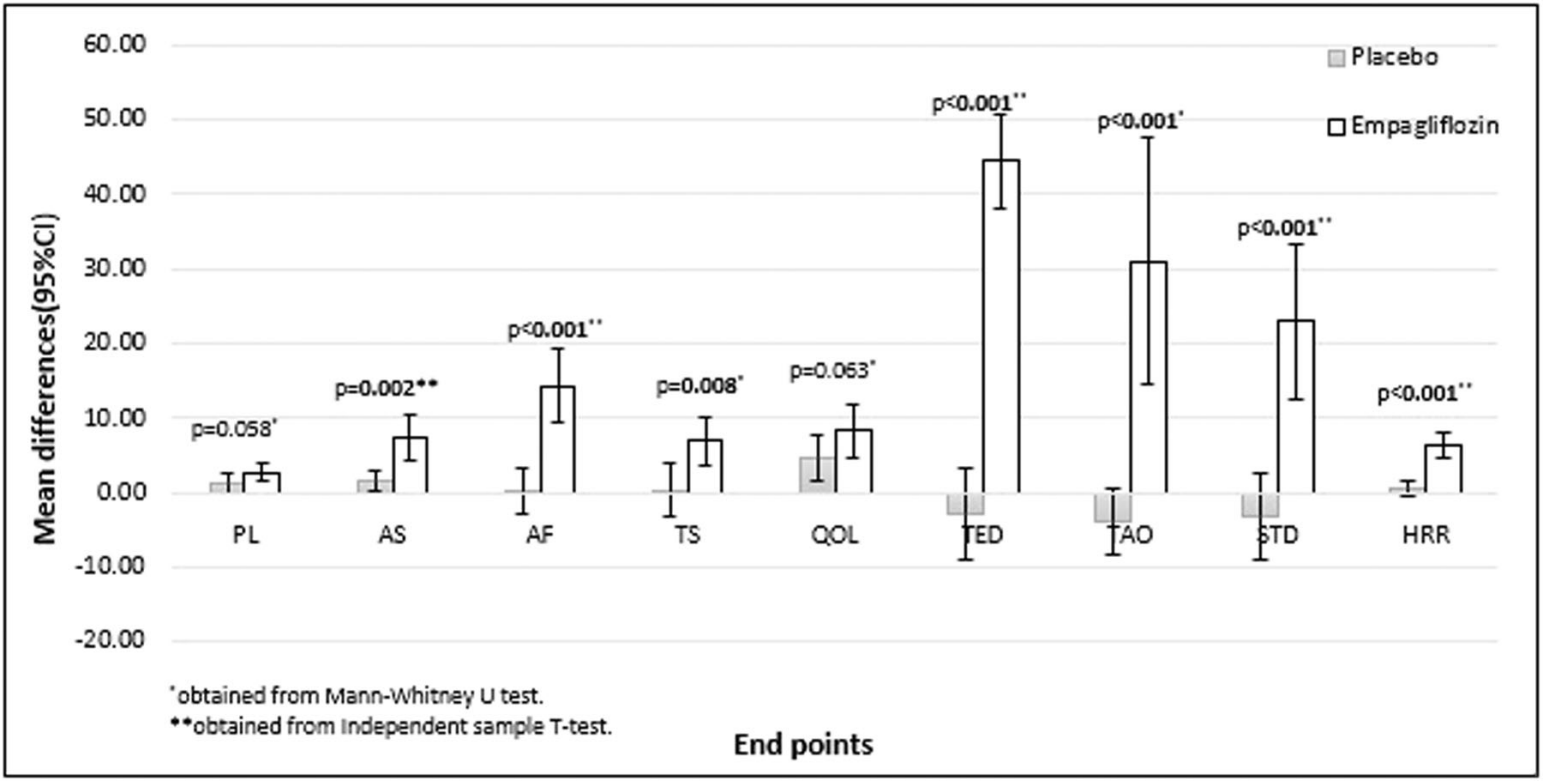
Mean changes (95% confidence interval [CI]) of endpoints (follow‐up minus baseline) in the treatment group.

Regarding secondary outcomes, there was no significant alteration in the placebo group except for QOL and AS, which were not significantly improved in comparison with the empagliflozin group after adjustment for baseline values. Adjusted models have also indicated the higher means of both primary and secondary endpoints in patients receiving Empagliflozin compared to the placebo group, except for PL. Figure 1 displays the mean changes (95% CI) of endpoints in the treatment group. As shown in Table 3, AS, AF, and TS showed clinically significant improvement in the empagliflozin group.

**Table 3:** A 10‐point change (clinically significant improvement) in SAQ domains.

	Empagliflozin (*n* = 37), *N* (%)	Placebo (*n* = 38), *N* (%)	*p* Value
PL	3 (8.1)	1 (2.6)	.297^a^
AS	15 (40.5)	1 (2.6)	<.001^b^
AF	22 (59.5)	2 (5.3)	<.001^b^
TS	14 (37)	3 (7)	<.001^b^
QOL	12 (32.4)	9 (23.7)	.399^b^

Abbreviations: AF, angina frequency; AS, angina stability; PL, physical limitation; QOL, quality of life; TS, treatment satisfaction.

^a^ Obtained from Fisher's exact test using Monte Carlo simulations.

^b^ Obtained from *χ* ^2^ test.

As shown in Table 4, no participant experienced any significant adverse events during the study time; however, mild side effects, including genital infection, increased urination, nausea, and increased thirst, were reported by patients in the empagliflozin group.

**Table 4:** Comparison of adverse side effects between treatment groups.

Adverse effect	Empagliflozin, *N* (%)	Placebo, *N* (%)	*p* Value
Non	31 (83.8)	37 (97.4)	.125^a^
Genital infection	1 (2.7)	0 (0)	
Increased urination	3 (8.1)	0 (0)	
Nausea	1 (2.7)	1 (2.6)	
Increased thirst	1 (2.7)	0 (0)	

^a^ obtained from Fisher's exact test using Monte Carlo simulations.

## 5 DISCUSSION

The EMPT‐ANGINA trial demonstrated that in individuals with concurrent T2DM and RA, adding empagliflozin to routine anti‐hyperglycemic therapies was linked to a substantial reduction in angina symptoms and improvement in exercise capacity compared to placebo.

Given the increased risk of cardiovascular complications in patients with T2DM, the cautious selection of antianginal treatment appears to be a crucial concern.

Antianginal therapy for diabetic patients with chronic stable angina appears to be a complex issue, partly because some of the pharmacological agents that alleviate angina may impair glucose regulation. In addition, unfavorable hemodynamic effects may make the patient less compliant.^3^ Therefore, it is crucial to find novel therapeutic measures that have both antianginal effects and glycemic control.^33^

SGLT2 inhibitors are novel, approved oral antidiabetic agents that block sodium and glucose cotransporters on the luminal surface of the proximal convoluted renal tubules.^34^ The resulting increase in natriuresis and glucose excretion leads to a wide range of metabolic benefits, such as a decrease in glycosylated hemoglobin,^35^ weight loss,^36^ decreased BP,^37^ anti‐inflammatory effects,^38^ decreased oxidative stress,^39^ reduced renin–angiotensin–aldosterone system activation^40^ and natriuretic peptides synthesis.^41^ Figures S2 and S3 represent possible mechanisms of cardiovascular benefits of SGLT2 inhibitors.

Myocardial ischemia is no longer considered a mere imbalance between myocardial oxygen demands and/or coronary blood flow to the ischemic myocardium but might be an issue related to energy metabolism.^42^ The heart mainly uses FAs as its energy source, but it can also use glucose and lactate. The normal cardiac cells have the ability to switch from FA to glucose when needed. This is called “myocardial flexibility.” T2DM disturbs this capability, and the myocardium becomes more dependent on FA oxidation for energy production. Furthermore, an increase in the delivery of FA due to peripheral insulin resistance leads to a decrease in glucose oxidation as a result of the Randle phenomenon and the activation of a nuclear receptor that regulates cellular FA metabolism, named peroxisome proliferator‐activated receptor‐α (PPAR‐α). Further inhibition of insulin signaling pathways by FA would also lead to reductions in glucose oxidation. Due to the low efficiency of FA versus glucose oxidation, less adenosine triphosphate (ATP) will be produced per each mole of oxygen used. This event leads to a fall in ATP content, high rates of glycolysis, pyruvate formation, and lactate accumulation, and a final decrease in intracellular pH and general disruption of cell homeostasis.^12^, ^25^, ^26^, ^43^, ^44^

Increased FA oxidation and hyperglycemia cause an accumulation of glucose and FA intermediate metabolites in the cells, resulting in lipotoxicity and glucotoxicity, respectively.^12^, ^25^, ^26^, ^43^ Ischemia‐induced cellular dysfunction can be minimized by metabolic agents that partially inhibit FA oxidation and increase the combustion rate of glucose and lactate. Agents inducing a switch in substrate use, reducing FA oxidation, and increasing glucose oxidation may have potentially beneficial effects on cardiac performance and decrease symptoms in patients with RA without alterations in the hemodynamic state.^12^, ^45^

Empagliflozin switches energy substrate preference from FA to glucose, resulting in the inhibition of cardiac FA oxidation and increasing the oxidation of pyruvate, leading to lower lactate production and less fall in myocytes' PH, which would be beneficial for the ischemic heart.^25^, ^26^ Metabolic effects of these agents might also play a role in ameliorating endothelial dysfunction with reduction of oxidative stress and inflammation as well as restoring nitric oxide (NO) bioavailability and enhancement of endothelium‐dependent vasodilation, which consequently improves coronary blood flow and microvascular function.^46^, ^47^

Large‐scale studies have shown that SGLT2 inhibitors enhance cardiorenal function and lower cardiovascular events, particularly heart failure.^48^, ^49^ In the EMPA‐REG OUTCOME study, empagliflozin outperformed placebo in terms of improving glycemic control and lowering CV events, including death, in patients with DM2 and established CV disease. There are several potential pathways for SGLT2 inhibitors' cardiovascular benefits, including afterload reduction, adequate BP control, and improving arterial stiffness.^50^, ^51^, ^52^ Even though there were no significant differences in our study's baseline and follow‐up HR and BP, the reduction in preload and myocardial stretch due to increased natriuresis may be responsible for the improvement of angina symptoms.^53^, ^54^ Another possibility for antianginal characteristics is an increase in hematocrit following the start of SGLT2 inhibitor medication.^55^

Our data demonstrated a substantial decrease in HRR following empagliflozin, indicating that SGLT2 inhibitors may have antisympatholytic effects that might relieve angina symptoms by lowering resting HR and cardiac demand.^56^, ^57^

## 6 LIMITATIONS AND SUGGESTIONS

To the best of our knowledge, this study was the first to assess the effect of empagliflozin on ischemic symptoms in patients with T2DM and RA. Although we tried our best to enhance the quality of this study, several limitations exist. Our sample size was relatively small, which might affect the generalization of our findings. We followed the patients for 8 weeks, which might be relatively short for an accurate assessment of the effectiveness of our intervention or the probable occurrence of adverse events. Furthermore, we did not measure the serum level of empagliflozin in the treatment group for further assurance of proper medication consumption.

## 7 CONCLUSION

The EMPT‐ANGINA trial showed that in people with concurrent T2DM and RA, adding empagliflozin to regular anti‐hyperglycemic treatments was linked to a significant decrease in angina symptoms and an increase in exercise capacity compared to placebo. These findings justify the use of empagliflozin in patients with DM and RA for clinical cardiologists.

## CONFLICT OF INTEREST STATEMENT

The author declares no conflict of interest.

## Supporting information

Supplementary Figure 1. Flow diagram of study.

Supplementary Figure 2. Probable mechanism of cardiovascular benefits of SGLT2 inhibitors. Hb, hemoglobin, HCT, hematocrit, BP, blood pressure.

Supplementary Figure 3. Probable anti‐anginal effect of empagliflozin, hemodynamic effect (A) metabolic effect (B).

## Data Availability

Derived data supporting the findings of this study are available from the corresponding author on request.
