# Supplementary figures and images for: Antianginal effects of empagliflozin in patients with type 2 diabetes and refractory angina; a randomized, double‐blind placebo‐controlled trial (EMPT‐ANGINA Trial)
Source: Clin Cardiol. 2023 Sep 18;47(1):e24158. doi: 10.1002/clc.24158 (PMC10766003; doi:10.1002/clc.24158)

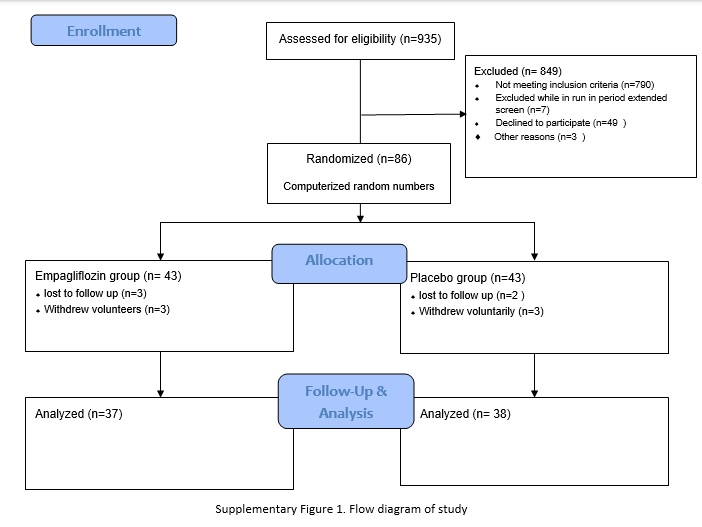

Supplement: Supplementary file 1 — Supplementary Figure 1. Flow diagram of study. [file CLC-47-e24158-s001.jpg]

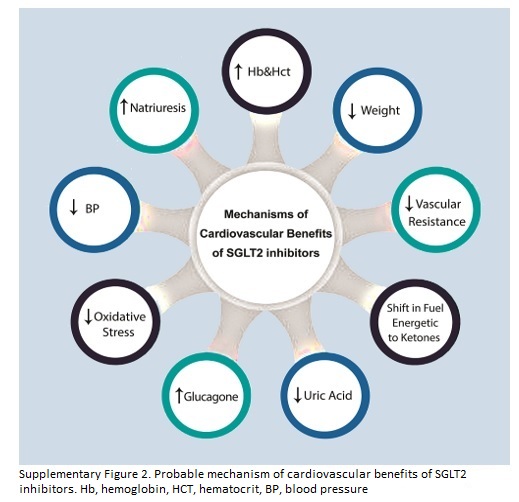

Supplement: Supplementary file 2 — Supplementary Figure 2. Probable mechanism of cardiovascular benefits of SGLT2 inhibitors. Hb, hemoglobin, HCT, hematocrit, BP, blood pressure. [file CLC-47-e24158-s003.jpg]

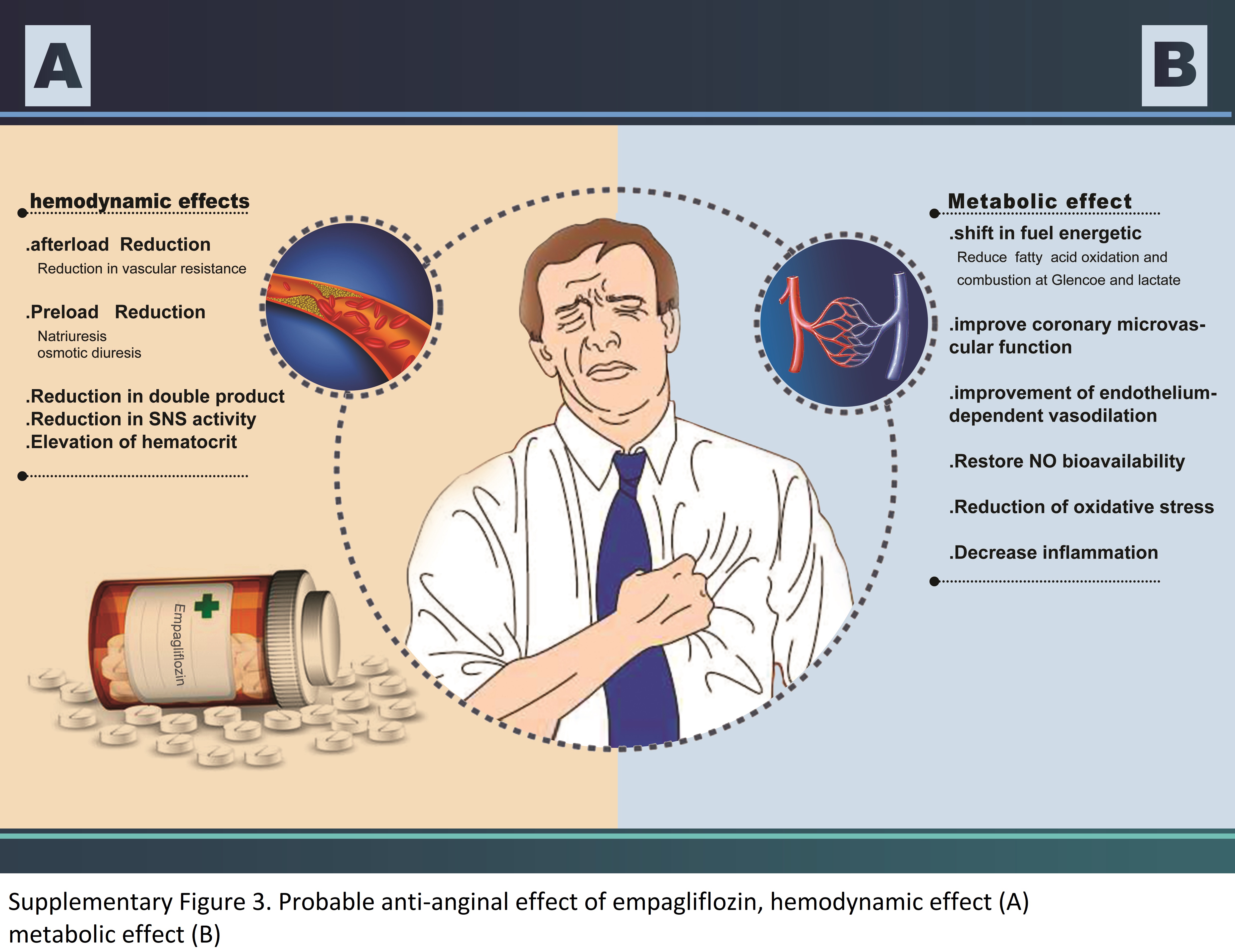

Supplement: Supplementary file 3 — Supplementary Figure 3. Probable anti‐anginal effect of empagliflozin, hemodynamic effect (A) metabolic effect (B). [file CLC-47-e24158-s002.jpg]
